# Humoral and cellular immune response over 9 months of mRNA-1273, BNT162b2 and ChAdOx1 vaccination in a University Hospital in Spain

**DOI:** 10.1038/s41598-022-19537-2

**Published:** 2022-10-07

**Authors:** Leire Fernández-Ciriza, Álvaro González, José Luis del Pozo, Alejandro Fernández-Montero, Francisco Carmona-Torre, Silvia Carlos, María del Mar Sarasa, Gabriel Reina

**Affiliations:** 1 Department of Microbiology, Clínica Universidad de Navarra, 31008 Pamplona, Spain; 2 Department of Biochemistry, Clínica Universidad de Navarra, 31008 Pamplona, Spain; 3 IdiSNA, Navarra Institute for Health Research, 31008 Pamplona, Spain; 4 Infectious Diseases Division, Clínica Universidad de Navarra, 31008 Pamplona, Spain; 5 Department of Occupational Medicine, Universidad de Navarra, 31008 Pamplona, Spain; 6 Department of Preventive Medicine and Public Health, Universidad de Navarra, 31008 Pamplona, Spain

**Keywords:** Vaccines, Viral infection, Viral infection

## Abstract

Scarce data have been reported about cellular immunity and longevity for different COVID-19 vaccination schedules. We carried out a prospective study enrolling 709 healthcare workers receiving two doses of mRNA-1273, BNT162b2, ChAdOx1, ChAdOx1/BNT162b2 or ChAdOx1 single dose to compare humoral and cellular immunogenicity across 9 months. Higher SARS-CoV-2 spike antibody levels were observed among individuals with hybrid immunity with one dose of any vaccine in comparison to uninfected individuals receiving two doses (mRNA-1273: 20,145 vs 4295 U/mL; BNT162b2: 15,659 vs 1959 U/mL; ChAdOx1: 5344 vs 2230 U/mL), except for ChAdOx1/BNT162b2 heterologous schedule (12,380 U/mL). Naturally infected individuals did not increase substantially the titers after the second dose and showed higher levels throughout the 9 months follow-up. The mean elimination half-life of antibodies among COVID-19 naïve participants was 98, 111, 60 and 36 days, for mRNA-1273, BNT162b2, ChAdOx1/ChAdOx1 and ChAdOx1/BNT162b2, respectively. Cellular immunity was preserved in 96%, 98%, 88% and 92% of uninfected individuals who received mRNA-1273, BNT162b2, ChAdOx1/ChAdOx1 and ChAdOx1/BNT162b2 after 6/9 months. Individuals with specific T cells showed robust long lasting protection, especially when m-RNA based vaccines are inoculated. These data may influence the validity of the vaccination passport and the need for booster vaccinations.

## Introduction

Coronavirus disease 2019 (COVID-19) has affected more than 530 million people and 6.3 million deaths have been recorded globally^1^ since the declaration of pandemics by the World Health Organization in March 2020. However, recent estimates suggest that 3.4 billion individuals or 44% of the global population, had been infected one or more times by SARS-CoV-2 virus before 2022^2^ and that 18.2 million people died worldwide following SARS-CoV-2 infection over that period^3^.

The rapid development of vaccines able to prevent new cases and decrease severity and lethality was imperative, leading to five COVID-19 vaccines approved in Europe based on mRNA, adenovirus or recombinant proteins: mRNA-1273 (Moderna), BNT162b2 (Pfizer-BioNtech), ChAdOx1 nCoV-19 (AZD1222; Oxford-AstraZeneca), Ad26.COV2.S (Janssen) and NVX-CoV2373 (Novavax)^4^. Soon after the authorization, vaccination campaigns across Europe began. So far, over 11.4 billion vaccine doses have been administered worldwide. These vaccines demonstrated remarkable efficacy and protection against mild and severe COVID-19 disease in clinical trials^5–7^. However, vaccine efficacy or effectiveness against infection and symptomatic disease decreased approximately 20–30 percentage points by 6 months, although protection against severe disease remained greater than 70% over time^8,9^. This reduction may be due to a loss of immunity, the circulation of new VOCs with the ability to evade the immune response, or a combination of both. In addition, some studies have shown that people who have undergone COVID-19 disease do not need a second dose if a first dose of mRNA vaccine has been administered^10^.

Specific T cells are detectable as early as 7–14 days post-vaccination or 3–5 days after onset of symptoms during natural infection, whereas binding and neutralizing antibodies are present at low titers at this early phase, and levels peak approximately at 14–21 days post-vaccination. Further data on the longevity of humoral and T-cell response is required to establish boosting schedules and measure the vaccine`s ability to recognize and protect against variants^11^.

Humoral and cellular immunogenicity of different homologous and heterologous combinations of mRNA and adenovirus-based vaccines have not been simultaneously compared during a long follow-up to assess the decline of each particular vaccine brand^12^. These data may be very useful when there are concerns around vaccine supply or vaccination passport validity, and further assessments are needed on the duration of the immune response, crucial for updating COVID-19 vaccine policies. We conducted a study in healthcare workers to understand the kinetics of humoral and cellular immunity during 6–9 months which could explain waning of vaccine-induced protection after vaccination with 5 different vaccine schedules.

## Results

Seven hundred and nine subjects were enrolled and subgrouped depending on the vaccine received (Table 1). The mean age of individuals was 44 ± 11 years old and 85.9% were women.

**Table 1:** Description of participants according to the type of vaccine inoculated.

	mRNA-1273/mRNA-1273	BNT162b2/BNT162b2	ChAdOx1/ChAdOx1	ChAdOx1/BNT162b2	ChAdOx1 single dose	Total
N	277	165	162	73	32	709
Age, mean (SD)	45 (10.9)	45.5 (11.9)	43.9 (10.7)	40.2 (10.4)	41.6 (12.0)	44.2 (11.2)
Women (%)	87.7	85.5	85.2	79.5	90.6	85.9
% Previous COVID-19	9.2%	43.1%	0.8%	0%	76%	17.2%
**Anti-S-RBD U/mL median (IQR) COVID-19 infected individuals**						
1 dose 21-days follow-up	20,145 (7450–27,473)	15,659 (10,448–22,027)	–	–	5344 (458–9550)	12,374 (6936–21,695)
2 doses^a^ 21-days follow-up	21,028 (9408–31,997)	21,433 (15,412–30,064)	–	3546 (968–4141)	3092 (1113–5353)	15,800 (4390–26,014)
2 doses^a^ 3-months follow-up	6711 (3814–12,119)	9800 (6631–15,948)	–	1544 (877–12,389)	2677 (1689–5769)	6902 (3098–11,256)
2 doses^a^ 6-months follow-up	3217 (1650–4781)	3900 (2664–6234)	–	1456 (404–8807)	1465 (799–3413)	3141 (1578–4985)
2 doses^a^ 9-months follow-up	1517 (851–3009)	2291 (1523–4958)	–	–	1222 (651–2342)	1926 (1041–4315)
**Anti-S-RBD U/mL median (IQR) COVID-19 non-infected individuals**						
1 dose 21-days follow-up	119.7 (49.9–193.9)	46 (17.4–96.6)	32 (13.8–68.2)		–	49.4 (22.1–123.2)
2 doses 21-days follow-up	4295 (2763–6203)	1959 (1221–3712)	2230 (1201–3667)	12,380 (8152–18,434)	–	3499 (1862–6185)
2 doses 3-months follow-up	2416 (1496–3736)	1336 (899.8–2065)	900 (570–1665)	3452 (2043–4616)	–	1758 (981.7–3173)
2 doses 6-months follow-up	1332 (970–2135)	805 (447–1118)	429 (272–757)	1370 (691–1882)	–	1004 (491–1595)
2 doses 9-months follow-up	674 (448–1093)	510,3 (267–758)	–	–	–	635 (397–971)
**% COVID-19 Infection (Anti-N > 0.150 COI) after 2nd dose**						
21-days follow-up	9.2%	43.1%	0.8%	0%	88%	18%
3-months follow-up	9.8%	42.2%	3.1%	0%	92%	19.3%
6-months follow-up	12.4%	43.1%	3.8%	0%	92%	20.5%
9-months follow-up	14.4%	42.2%	–	–	92%	31.9%

^a^2 doses of mRNA-1273, BNT162b2, ChAdOx1/ChAdOx1 or ChAdOx1/BNT162b2 schedules or one single dose of ChAdOx1 vaccine.

### Humoral immunity

**Figure 1:**
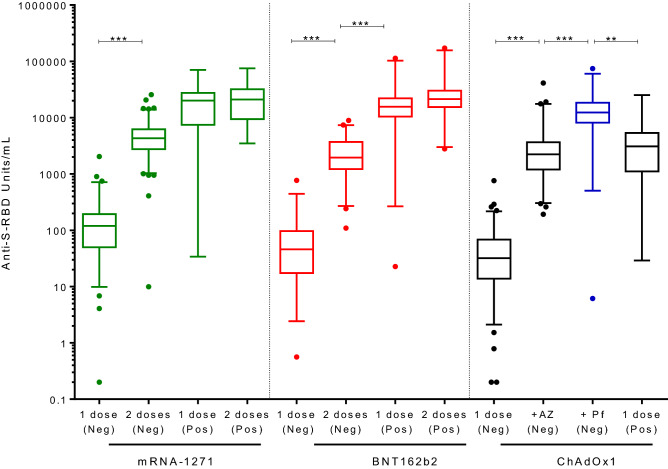
Anti-S-RBD (U/mL) levels after administration of one or two doses (21 days follow-up). mRNA-1273 (green), BNT162b2 (red) or ChAdOx1 (black/blue) vaccines among individuals with previous COVID-19 infection (Pos) or not (Neg) (ChAdOx1/ChAdOx1 (AZ): black; ChAdOx1/BNT162b2 (Pf): blue) (**p < 0.01; ***p < 0.001).

Significant differences were found in all vaccines subgroups in the production of anti-RBD antibodies after receiving one or two doses in those individuals who had not undergone previous COVID-19 infection. However, no differences were found between individuals with a history of COVID-19 within the mRNA-1273 and BNT162b2 groups, after receiving the second dose (Fig. 1).

The inoculation of one dose of BNT162b2 or ChAdOx1 resulted in similar levels of production of anti-RBD antibodies among COVID-19 naïve individuals, while one shot of mRNA-1273 showed a higher level (p < 0.05). In contrast, after two doses among participants not previously infected by SARS-CoV-2, the schedule ChAdOx1/BNT162b2 showed the highest level of antibody production followed by mRNA-1273, BNT162b2 or ChAdOx1/ChAdOx1 schedules (p < 0.05).

Higher antibody titers were observed in previously infected participants vaccinated with one dose of mRNA-1273 or BNT162b2 (median titer, 20,145 U/mL (IQR, 7450–27,473) and 15,659 U/mL [IQR, 10,448–22,027, respectively, measured 21 days after the first shot) compared with those COVID-19 naïve individuals vaccinated with two doses (median titer, 4295 U/mL (IQR, 2763–6203); 1959 U/mL (IQR, 1221–3712), respectively, 21 days after their second shot) (p < 0.001). In contrast with other vaccines, when ChAdOx1 schedules were analyzed, a single dose received by those who had undergone COVID-19 yielded higher levels of Anti-S-RBD (5344 U/mL (IQR, 458–9550) than the ChAdOx1/ChAdOx2 two dose schedule of COVID-19 naïve participants (2230 U/mL (IQR, 1201–3667)), but lower than the ChAdOx1/BNT162b2 combination (12,380 U/mL (8152–18,434)) (Fig. 1).

**Figure 2:**
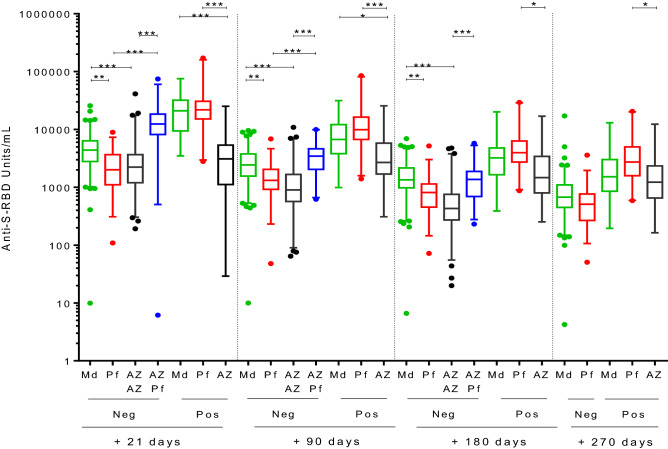
Anti-S-RBD (U/mL) kinetics over different vaccine schedules. Anti-S-RBD (U/mL) kinetics over 9-months follow-up of mRNA-1273 (Md) (green), BNT162b2 (Pf) (red) and ChAdOx1 (AZ) single dose schedule (black). Anti-S-RBD (U/mL) kinetics over 6-months follow-up of first dose ChAdOx1 vaccine and second dose (ChAdOx1 (black) or BNT162b2 (blue) (Md: mRNA-1273; Pf: BNT162b2; AZ: ChAdOx1) (*p < 0.05; **p < 0.01; ***p < 0.001).

Similar differences were found between vaccines after 21 and 90 days after the second dose, mRNA-1273 and ChAdOx1/BNT162b2 were the most highly immunogenic schedules among COVID-19 naïve individuals, and ChAdOx1/ChAdOx1 yielded the lowest level of Anti-S-RBD up to 6 months after the second dose. Those individuals vaccinated with mRNA vaccines (2 doses) and had undergone COVID-19 infection, were able to maintain higher levels of Anti-S-RBD than those vaccinated with a single dose of ChAdOx1 vaccine during the first 3 months of follow-up (Fig. 2).

At 6 months follow-up, no significant differences were observed between BNT162b2 and ChAdOx1/BNT162b22 combinations among naïve participants, neither between both m-RNA vaccines after 9 months within this group. Analyzing the results from participants with previous COVID-19, significant differences were found between BNT162b2 and ChAdOx1 single dose vaccination during all the follow-up period, while no differences were observed between mRNA-1273 (2 doses) and ChAdOx1 single dose at the end of the 9-months follow-up.

**Figure 3:**
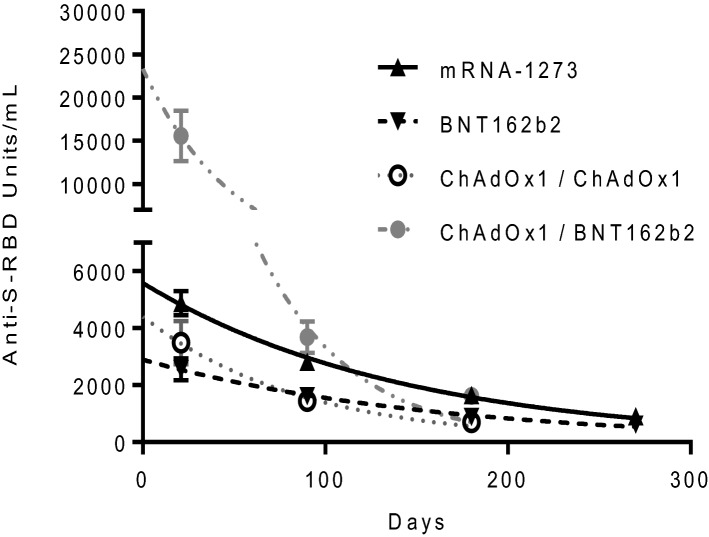
Antibody kinetics among naïve participants. Anti S-RBD kinetics among participants without previous COVID-19 infection receiving two doses of mRNA-1273, BNT162b2, ChAdOx1/ChAdOx1 or ChAdOx1/BNT162b2 vaccines.

The elimination half-life of anti-S-RBD antibodies produced in persons who had received two doses of the mRNA-1273 and BNT162b2 vaccines was 98 and 111 days, respectively. However, the half-life of anti-S-RBS antibody concentration in those participants who received a first dose of ChAdOx1 was 36 and 60 days, depending on the second inoculated dose, BNT162b2 and ChAdOx1, respectively (Fig. 3).

### Cellular immunity

**Figure 4:**
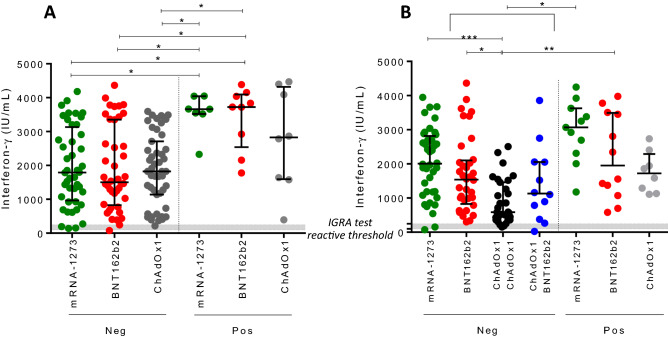
IFN-γ production levels among previous COVID-19 infection (Pos) and not (Neg). Cellular immunogenicity (IFN-γ levels after specific stimulation) of individuals: (**A**) 21 days after dose 1; (**B**) 9 months after mRNA-1273 or BNT162b2 homologous second dose, and ChAdOx1 single dose; 6 months after ChAdOx1/ChAdOx1 or ChAdOx1/BNT162b2 second dose (*p < 0.05; **p < 0.01; ***p < 0.001).

The cellular response was studied in 51 individuals of group 1, 51 of group 2 and 56 of groups 3/4/5. The cellular immunity after one dose was positive for 94.1%, 96.1% and 98.2% participants who had received mRNA-1273, BNT162b2 or ChAdOx1 vaccines, respectively. All those with a previous infection showed the presence of specific T-cells and the stimulation yielded higher levels of IFN-γ when any m-RNA based vaccine was administered (p < 0.05) compared to COVID-19 naïve individuals. The level of IFN-γ production among naïve individuals was similar for the three vaccines (Fig. 4).

Nine-months after second dose the cellular immunity was preserved in 96.1% and 97.9% of uninfected individuals who had received mRNA-1273 and BNT162b2 vaccines, respectively. For those who received ChAdOx1/ChAdOx1 or ChAdOx1/BNT162b2 combinations, cellular immunity was found in 88.2% and 92.3%, respectively, after 6-months among COVID-19 naïve participants. Again, all tests to assess cellular immunity among individuals with a previous infection were reactive.

The level of cellular immunity was higher for participants who received two doses of mRNA-based vaccines, and particularly the schedule ChAdOx1/ChAdOx1 among COVID-19 naïve individuals showed the lowest level of IFN-γ production after stimulation. No differences were found between ChAdOx1/ChAdOx1 and ChAdOx1/BNT162b2 schedules (Fig. 4).

Hybrid immunity among those vaccinated individuals with any m-RNA vaccine who had undergone COVID-19 infection showed a more robust and long-lasting cellular immunity (IFN-γ levels were significantly higher at the end of the follow-up period). No significant differences were observed between participants with or without prior COVID-19 vaccinated with mRNA-1273. There was also no inferiority of IFN-γ production among those participants with a previous COVID-19 infection who received the single dose ChAdOx1 schedule, compared to the rest of schedules in the study.

## Discussion

This study demonstrated a significantly higher humoral immunogenicity with one single dose of either SARS-CoV-2 vaccine among those individuals infected by SARS-CoV-2 virus in comparison to uninfected individuals after receiving two doses, except for those who got the heterologous combination of ChAdOx1 (first dose) and BNT162b2 (second dose). A single-dose schedule of mRNA-based vaccines (mRNA-1273 or BNT162b2) has been reported to elicit a rapid immune response in seropositive individuals with similar or higher antibody titers than seronegative who received two doses^13^. We observed that the second dose of mRNA-1273 or BNT162b2 vaccines inoculated to previously infected individuals did not increase substantially the level of RBD-specific antibodies, as suggested by other authors^14^.

For those participants with a hybrid immunity from ChAdOx1 single dose vaccine and natural infection, no differences were found after 3 and 6 months follow-up in comparison to uninfected individuals who had received ChAdOx1/BNT162b2 combination. It has been previously reported that post-vaccine levels of RBD-specific IgG and neutralizing antibodies against the SARS-CoV-2 were similar or higher in participants receiving a single dose of ChAdOx1 vaccine after natural infection compared to uninfected individuals who received two doses of BNT162b2 vaccine^15^.

Throughout the 9 months follow-up, previously infected participants who received mRNA-1273 or BNT162b2 vaccines resulted in significantly higher levels of RBD-specific antibodies in comparison to uninfected individuals. In addition, mRNA-1273 and ChAdOx1/BNT162b2 schedules among COVID-19 naÏve participants showed the highest levels of Anti-S-RBD antibodies during the follow-up. Among mRNA-based vaccines, the higher mRNA content in mRNA-1273 compared with BNT162b2 and the longer interval between priming and boosting for mRNA-1273 (4 weeks vs 3 weeks) might explain this difference^16^. It is known that the level of the humoral response after vaccination correlates with neutralizing antibody titers^17^ which might be clinically relevant and decrease to a marked extent the risk of reinfection^16^. In addition, the importance of additional antibodies that induce neutrophil phagocytosis and natural killer cell activation has been highlighted with the emergence of viral variants able to evade vaccine-induced neutralizing antibodies. These non-RBD-specific antibodies with Fc-mediated effector functions have been reported to be increased among mRNA-1273 vaccine recipients compared to BNT162b2^18^.

The participants receiving mRNA-based vaccines showed a half-life of antibody levels above 90 days, which eventually leads to anti-S-RBD median levels of 674 U/mL and 510 U/mL, for mRNA-1273 and BNT162b2, respectively, among uninfected participants after 9 months; and 1517 U/mL and 2291 U/mL, respectively, among previously infected individuals. These data are above the previously reported half-life of 68 days for neutralizing antibodies induced by BNT162b2 vaccination^19^. The analysis of S-RBD antibodies when first dose of ChAdOx1 vaccine was administered resulted in levels of 429 U/mL and 1370 U/mL among uninfected individuals, if ChAdOx1 or BNT162b2 vaccine, respectively, was inoculated as second dose. However, the half-life for both vaccination schedules receiving a first dose of ChAdOx1 vaccine was much shorter (36 or 60 days, if a second dose of BNT162b2 or ChAdOx1 vaccine, respectively, was inoculated). The differences among antibody decay are influenced by the different vaccine schedules and may be due to different reasons such as the tools used to measure them or the age, sex and health status of participants.

The induction of specific T cells with effector capacity and immunological memory is essential to reduce the symptoms and severity of COVID-19. In addition, its protective effect can last for years and does not depend on the infecting VOC, since T cells can recognize further sites than antibodies within the spike protein and there is a cross-protective effect induced by different coronaviruses^20^. Even the Omicron variant, which has multiple spike mutations that contribute to viral escape from neutralization, thus reducing protection against infection, may be a target for T cells. Different vaccines schedules and COVID-19 convalescent unvaccinated individuals maintain a high presence of CD4+ and CD8+ against Omicron Spike and the magnitude of cross-reactive T cells is similar for the Omicron, Beta and Delta variants^21^.

Every participant with hybrid immunity showed specific cellular immunity 21 days after the first dose and 9 months after receiving the two dose schedule of mRNA-based vaccines or 6 months after a single dose of ChAdOx1, consistent with the fact that cellular immune response is detected in convalescent individuals after moderate, mild, or completely asymptomatic COVID-19 at least 200 days after infection^22,23^ and thus, the development of medium or long-term protective immunity following vaccination is possible. Recent reports of large cohorts have shown that naturally acquired immunity confers equal or even superior protection against infection and symptomatic disease, compared to vaccine-induced immunity^24,25^. Also, previous studies on the original SARS-CoV have shown that memory cells can be detected up to 17 years after infection^20^.

Among naïve participants, most vaccinees also developed and maintained cell-mediated immunity, but the level of IFN-γ following stimulation was higher for participants with prior COVID-19 infection, particularly when mRNA-1273 or BNT162b2 were used, confirming previous data which, in addition, showed that vaccine-induced T cell response is largely conserved against viral variants^26,27^. Considering the different vaccines, the homologous schedule ChAdOx1/ChAdOx1 got the lowest level of IFN-γ production suggesting that this combination was less effective as recently highlighted, before and after second dose^28^.

The heterologous schedule ChAdOx1/BNT162b2 demonstrated significantly greater Anti-S-RBD levels than any other combination, but the decay was also greater. However, the titers were similar to those generated by the homologous BNT162b2/BNT162b2 vaccination and permanently higher than the homologous ChAdOx1/ChAdOx1 dosing, as reported before^29^. Considering the cellular immunity created by the heterologous schedule ChAdOx1/BNT162b2, we could see a level of cytokine-producing T cells similar to that induced by BNT162b2, improving the immunity induced by the homologous ChAdOx1/ChAdOx1 schedule, as described previously^30,31^.

Our study is subject to a number of potential limitations. The first is that participants with a history of COVID-19 received two doses of m-RNA based vaccines, so no information of a single dose regimen for those vaccines could be obtained. Second, cellular immunity was measured at two single points, but an overview of cell-mediated immunity could be obtained from the beginning and the end of the follow-up period. In addition, pre-infection rate was higher in BNT162b2/BNT162b2 group, due to the availability of vaccines during the vaccination campaign; for this reason, the results are given by subgrouping the data according to previous infection and the evaluation of cellular immunity was performed after making a homogeneous selection of participants for the different vaccine schedules in terms of age, sex and previous natural infection. Finally, we had an unbalanced sex distribution, and a mostly middle-aged population, which limited the precision of the estimates. Age-associated immune system dysfunction, manifested by altered immune parameters such as decreased lymphocyte function or rapid antibody clearance, may eventually predispose to severe COVID-19; therefore, a booster vaccine offers great benefits in the elderly population to maintain immunity, especially if vaccines are reformulated to improve the production of specific antibodies against new variants such as Omicron^32–34^.

The study has several notable strengths, including the use of standardized and commercial IGRA and validated robust serological platforms to measure values of interest for the study. To our knowledge this is the largest study to evaluate humoral and cellular immunity for nine months produced after receiving 5 different schedules of vaccination among naïve individuals and others with a previous infection. This is also the first time that the half-life of Anti-S-RBD antibodies generated by different vaccination schedules has been established and additional questions arise, since the European vaccination passport may have different validity depending on whether individuals have undergone COVID-19 or not, or depending on the vaccine received.

In conclusion, this study confirms improved immunogenicity after 9 months of m-RNA based vaccines inoculated using both homologous and heterologous dosing. In addition, a robust hybrid immunity can be obtained using a single vaccine dose as individuals with specific T cells got little benefit from booster shots.

## Material and methods

### Study design and participants

A prospective cohort study was performed among healthcare workers of Clinica Universidad de Navarra in Pamplona (Spain), during the SARS-CoV-2 pandemic, from March 2021 to December 2021 (SAVIN Project: SARS-CoV-2 Vaccine Immunity Navarra).

All the staff who were going to receive full coverage of mRNA-1273, BNT162b2 and ChAdOx1 vaccines were invited to participate voluntarily in the study. Different vaccination schedules were used depending on availability, age or contact with patients. Seven hundred and nine subjects were finally enrolled and subgrouped depending on the vaccine received (group 1: mRNA-1273 vaccine; group 2: BNT162b2 vaccine; groups 3/4/5: ChAdOx1 nCoV-19 as first dose vaccine). The humoral response was analyzed in all the participants (n = 709) and the cellular response in 158 volunteers, making a homogeneous selection of participants for the different vaccine schedules in terms of age, sex and previous natural infection. Immunogenicity was compared in individuals with and without documented pre-existing SARS-CoV-2 immunity. Demographics were recorded from the hospital register.

For mRNA-1273 and BNT162b2, the vaccination schedule included 2 doses for all participants (previously infected and non-infected individuals) separated by 28 and 21 days, respectively. For those who got a first dose of ChAdOx1 vaccine and had not been previously infected, they received a second dose of ChAdOx1 (group 3) or BNT162b2 (group 4) vaccine at free choice, 12 weeks after the first shot. Individuals previously infected and vaccinated with a first dose of the ChAdOx1 vaccine did not receive any second dose (group 5).

Heparin whole blood and plasma samples were obtained from every volunteer. Serological analyses were performed in plasma specimens while whole blood samples were used to study the cellular immune response.

**Figure 5:**
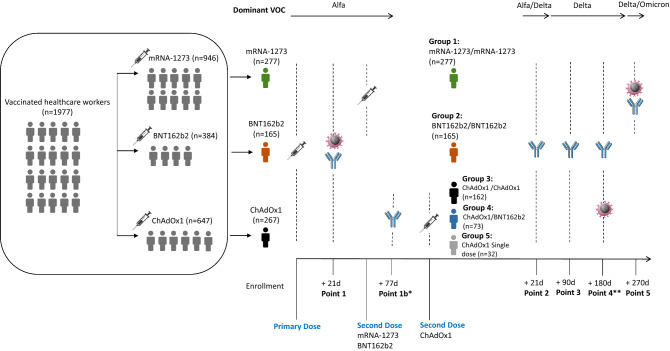
Flowchart of SAVIN participants recruited and follow-up summary for humoral and cellular response evaluation (*Point 1b (before second dose): humoral response evaluation of Groups 3/4/5; **Point 4: cellular response evaluation of Groups 3/4/5 and humoral response evaluation of all groups). Source of icons: lymphocyte (www.cleanpng.com), antibody (view.genial.ly), syringe (www.istockphoto.com), person (www.flaticon.es). Image drawn by LFC, SC and GR.

Samples were collected in order to monitor the immune response of each vaccination schedule at five time points, starting 21 days after the first dose (point 1) and continuing 21 days (point 2), 90 days (point 3), 180 days (point 4) and 270 days (point 5) after the second dose. For those participants who received the vaccination schedule with mRNA-1273 or BNT162b2, samples 1–5 were collected, completing 9 months post vaccination follow-up. In subjects who received ChAdOx1 vaccine as first dose, an extra sample (point 1b) was collected between the first and the second dose due to the time difference between them (77 days after first dose), and the last sample of follow-up collected was at point 4, completing a 6 months period follow-up after the second dose. The cellular response was studied at points 1 and 5, whereas the humoral response was studied at the five points of sampling (Fig. 5).

### Humoral response evaluation

Anti-SARS-CoV-2 antibody detection was performed using two different commercial chemiluminescence tests. First, quantification of total antibodies (IgG + IgM) against the receptor binding domain (RBD) of SARS-CoV-2 spike (S) protein was performed, using Elecsys® Anti-SARS-CoV-2 S (Roche Diagnostics, Germany) test in the cobas e601 platform. Second, qualitative detection of total antibodies (IgG + IgM) against viral nucleocapsid (Anti-N) was done using Elecsys® Anti-SARS-CoV-2 test (Roche Diagnostics, Germany). Anti-S-RBD levels were quantified and considered reactive or non-reactive using a cut-off level of 0.8 U/mL. Anti-N official cutoff index (COI) supplied by the manufacturer is 1.0; however, as this was a follow-up study and after optimization of the COI value, Anti-N results were interpreted as reactive (previous natural infection) or non-reactive using a COI value of 0.150.

### Cellular response evaluation

Cell-mediated immune response to SARS-CoV-2 was measured using SARS-CoV-2 IGRA Test (Euroimmun, Germany) using fresh blood specimens processed at less than 8 h from venipuncture. To carry out the test, 0.5 mL of blood was inoculated in three different tubes. Two tubes to ensure an appropriate immune cellular response and to determine the individual interferon-gamma (IFN-γ) background, and a third tube coated with components of the S1 domain of the SARS-CoV-2 spike protein to study an active specific cellular response. After inoculation, the tubes were incubated for 20–24 h at 37 °C, and subsequently IFN-γ concentrations were measured in plasma by ELISA. Samples were considered non-reactive when IFN-γ production following S1 stimulation was below 100 IU/mL, borderline if the concentration was 100–250 IU/mL and reactive if the level was above 250 IU/mL. This IGRA test is a reliable method for quantifying T-cell response after SARS-CoV-2 vaccination or infection, with overall sensitivity and specificity (95% confidence interval) of 81.1% (74.9–86%) and 90.9% (74.5–97.6%), as previously reported^35^.

### Ethical statement

The planning, conduct and reporting of the studies was in line with the Declaration of Helsinki. The project was approved by the Human Subjects Review Committee for Clinical Research of Navarra (Pamplona, Spain) (Reference: EO_2021/6. Code: SAVIN). Informed consent of enrolled participants was obtained. All methods were carried out in accordance with relevant guidelines and regulations (CPMP/ICH/135/95).

### Statistical analysis

Due to their non-normal distribution determined with the Shapiro-Wilks tests, concentrations were expressed as median and Interquartile range (IQR). The non-parametric Mann–Whitney U test, Kruskal–Wallis test and Friedman test with Dunn’s multiple comparison tests were applied to assess the statistical differences in antibody and IFN-γ levels. Correlation analysis was performed using the Spearman correlation test. Antibody concentration decay was calculated using the one phase exponential decay formula: Yt = Y0 × e exp(− K × t) where Yt is the antibody concentration at time t (days), and Y0 is the antibody concentration 21 days after the second dose.

Statistics and graphs were obtained using Stata 15.0. (https://www.stata.com) and GraphPad Prism 5 Software (https://www.graphpad.com/scientific-software/prism). A two-tailed P-value < 0.05 was considered statistically significant.

## Data Availability

The datasets generated and/or analysed during the current study are available in the Harvard Dataverse Repository, 10.7910/DVN/KOWTIC.
